# Extracellular Vesicles in Pulmonary Fibrosis Models and Biological Fluids of Interstitial Lung Disease Patients: A Scoping Review

**DOI:** 10.3390/life11121401

**Published:** 2021-12-15

**Authors:** Miriana d’Alessandro, Laura Bergantini, Elena Bargagli, Silvia Vidal

**Affiliations:** 1 Respiratory Diseases and Lung Transplant Unit, Department of Medical and Surgical Sciences & Neurosciences, University of Siena, 53100 Siena, Italy; laurabergantini@gmail.com (L.B.); bargagli2@gmail.com (E.B.); 2 Inflammatory Diseases, Institut de Recerca de l’Hospital de la Santa Creu i Sant Pau, Biomedical Research Institute Sant Pau (IIB Sant Pau), 08041 Barcelona, Spain; svidal@santpau.cat

**Keywords:** extracellular vesicles, interstitial lung disease, pulmonary fibrosis

## Abstract

Introduction: Interstitial lung diseases (ILDs) are a heterogeneous group of diffuse parenchymal lung disorders characterized by the pathogenetic involvement of interstitium. Therefore, an elucidation of the etiology and pathogenesis as well as the identification of diagnostic and prognostic biomarkers of such diseases is more compelling than ever. It is of note that there is increasing evidence of the involvement of extracellular vesicles (EVs) in the pathogenesis of lung diseases including lung cancer, chronic obstructive pulmonary disease and pulmonary fibrosis. It has been speculated that EVs play a pivotal role as mediators of intercellular communication, as well as the highlighting of the role of EVs as co-operators in the development of lung diseases such as IPF. Methods: The present study aimed to carry out a systematic exploratory search of the literature (through the scoping review approach) to identify and systematize the main results of the pathogenetic role of EVs in pulmonary fibrosis models and biological fluids from ILD patients, including plasma, bronchoalveolar lavage (BAL) and sputum. Conclusion: Fibroblast-to-mesenchymal differentiation, collagen and extracellular matrix deposition are key mechanisms in the development and progression of IPF. EV-coupled miRNA are important modulators of biological processes in terms of intercellular communication as shown in pulmonary fibrosis models as well as biofluids. The helpfulness of EVs as diagnostic and theranostic markers is worth further investigation. The evolving potential of EVs to translate effective EV-based therapies into clinical practice is of growing interest, due to the urgent need for novel therapeutic strategies for IPF patients.

## 1. Introduction

Interstitial lung diseases (ILDs) are a heterogeneous group of diffuse parenchymal lung disorders characterized by the pathogenic involvement of the interstitium [1]. Many ILDs are of unknown cause and are termed idiopathic interstitial pneumonia (IIP); the most frequent form of IIP is idiopathic pulmonary fibrosis (IPF), which has a progressive clinical course, sometimes interrupted by events associated with high mortality, defined as “acute exacerbations” [2]. IPF has a poor prognosis: median survival is 4 to 5 years from the time of diagnosis [2].

In recent years, it has also been reported that patients with non-IPF ILDs, such as non-specific interstitial pneumonia [3], fibrotic hypersensitivity pneumonitis [4] and connective tissue diseases with ILD [5], can show progressive worsening of the disease regardless of treatment. Many recent studies also suggest that IPF and non-IPF ILDs have common pathogenetic pathways, like the shortening of telomeres, epithelial cell dysfunction and immune dysregulation [6,7]. For these reasons, a new progressive fibrotic phenotype, based on clinical and functional progression of the disease, was recently proposed to include all ILD patients (both idiopathic and non-idiopathic) who show inexorable deterioration [8]. This makes the elucidation of the aetiology and pathogenesis, as well as identification of diagnostic and prognostic biomarkers of such diseases, more compelling than ever.

There is increasing evidence of the involvement of extracellular vesicles (EVs) in the pathogenesis of lung diseases, including lung cancer, chronic obstructive pulmonary disease and pulmonary fibrosis. It has been speculated that EVs play a pivotal role as mediators of cell–cell communication, as well as having a co-operator role in the development of lung diseases such as IPF [9]. ‘Extracellular vesicles’ is a generic term for particles released naturally by cells. They are enclosed in a lipid bilayer and cannot replicate (i.e., do not contain a functional nucleus) [10]. No consensus has yet emerged on specific markers of EV subtypes with particular biogenetic pathways, such as “exosomes” arising from endosomes, plasma membrane-derived “ectosomes” (microparticles/microvesicles) and apoptotic bodies [10].

Standardized recovery of EVs from an EV-containing matrix is still challenging and many questions remain about the effects of specific pre-analytical variables on different classes of EVs. This may be due to the fact that mammals have more than 30 types of biofluids. To these we can add lavages of various compartments, which are not true biofluids.

Extracellular vesicles are secreted by a wide variety of cells under physiological and pathological conditions and have been described as important mediators of intercellular communication in different pathologies. They carry a wide variety of bioactive molecules such as DNA, RNA, lipids and proteins, which enable them to regulate host cell activity and behaviour by activating different signalling pathways [10]. Mechanistically, EVs have been shown to function in most, if not all steps of pulmonary fibrosis models. However, the role of EVs and their translational applications in fibrotic ILD patients is relatively unexplored, despite their likely potential as diagnostic and theranostic markers of fibrotic ILDs.

The aim of the present study was to conduct a systematic exploratory search of the literature (by a scoping review approach) to obtain a systematic picture of what we know about the pathogenetic role of EVs in pulmonary fibrosis models and biological fluids from ILD patients, including plasma, bronchoalveolar lavage (BAL) and sputum.

## 2. Methods

We used the scoping review protocol [11] and descriptive thematic analysis, later detailed, to piece together the involvement of EVs in the pathogenesis of ILDs (mainly IPF). This article conforms to the guidelines of the Scale for Assessment of Narrative Review Articles (SANRA) [12].

### 2.1. Eligibility Criteria 

The inclusion criteria were peer-reviewed, empirical or perspective papers (including editorials and commentaries) with: (a) relevance to the study topic, i.e., involvement of EVs in pulmonary fibrosis studied in different biological fluids from ILD patients, as well as in in vitro and in vivo models; (b) language: English; (c) type of journal: preference for journals relating to pneumology with full text or abstract; d) type of study: review, case report, case series, original article or letter to the editor. Studies were excluded if: (a) they were not relevant to the topic of study; (b) they were written in languages other than English; (c) they did not adequately report their objectives and conclusions; (d) the full text of abstracts located was not (yet) available.

### 2.2. Information Sources and Search

A systematic search of the literature was conducted in the PubMed online database. The term we entered in our Boolean search syntax was: extracellular vesicles or exosomes AND (“interstitial lung disease” OR “pulmonary fibrosis”). As already mentioned, the search was limited to the English language and papers available in full text form. We did not search the grey literature (e.g., official reports from international organizations). 

### 2.3. Selection Process 

Two independent reviewers (M.d., L.B.) screened the abstracts and titles, and ascertained the availability of the full-texts. If the papers met the eligibility criteria they were considered. Any disagreement of the reviewers was resolved by consensus or by the leading reviewer (M.d.). 

### 2.4. Data Charting and Items 

**Figure 1:**
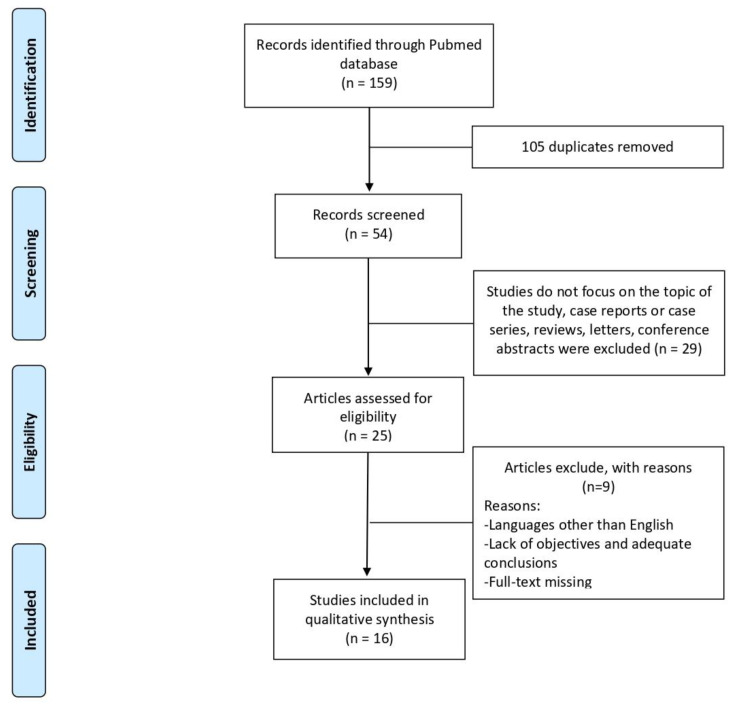
Flowchart of included articles for the scoping review.

Using a coding structure devised by members of the research team, one author (M.d.) extracted formal data items (publication type, sources, geographies addressed, objectives and main findings) and a random 5% were checked by another (L.B.). Regarding the content of the literature included, three independent reviewers (M.d., E.B., S.V.) extracted text quotations on: (1) analysis of EVs in lung fibrotic models and their involvement in the pathogenesis of pulmonary fibrosis, or (2) detection of EVs in sputum, BAL and serum from ILD patients. These independent extractions were later paired for qualitative data synthesis, which was also supplemented with brief summaries of each paper made independently by the two reviewers. The extractions and summaries of all reviewers were then combined. Figure 1 shows the selection flowchart.

## 3. Results

### 3.1. Synthesis of the Results—Simple Descriptive Data 

The literature search yielded all articles concerning “extracellular vesicles”, an umbrella term that covers all vesicle types, including “exosomes” and “microvesicles” (historically burdened with contradictory definitions and the unrealistic expectation of a single biogenetic process) generated by inward budding of the cell membrane (endocytosis), subsequent shaping of multivesicular bodies and release by exocytosis. During the release into the extracellular space, the peripheral membrane of these multivesicular bodies fuses and the exosomes incorporate various membrane proteins, including tetraspanins (CD9, CD63, CD81). These surface proteins are recognized as markers of exosomes. Of the 16 studies selected, three included the possible involvement of EVs in pulmonary fibrosis models, three speculated that EVs could be a potential new target for clinical treatment of lung diseases, four analysed BAL EVs in the pathogenesis of ILDs, two concerned the role of EVs from sputum as potential biomarkers of IPF and three suggested a possible role of EVs as mediators and disseminators of inflammation in ILD patients. 

### 3.2. Quality Assessment after SANRA

The results of SANRA are reported in Table 1. All 48 ratings (3 raters × 16 manuscripts) were used for statistical analysis. The mean sum score across all 16 manuscripts was 8.067 out of 12 possible points (SD 0.884, range 7–9, median 8). High scores were rated for item 1 (Justification of the article’s importance for the readership) (mean 1.8; SD 0.414), item 2 (Statement of concrete aims or formulation of questions) (mean 1.667; SD 0.488), item 5 (Scientific Reasoning) (mean 1.733; SD 0.457) and item 6 (Appropriate presentation of data) (mean 1.6; SD 0.507), whereas item 4 (referencing) had the lowest score (mean 1.267; SD 0.458). We did not evaluate item 3 (Description of the literature search) because we did not consider any kind of review.

### 3.3. EVs in Pulmonary Fibrosis Models

The formation of fibroblastic foci, excessive deposition of extracellular matrix proteins cellular senescence and senescence-associated secretory phenotype factors are key factors that directly cause IPF; however, the underlying mechanisms are still unclear [1,2,13]. Because there are no natural models that sum up all features of IPF, the use of animal models that reproduce known key hallmarks of the disease is warranted. One of the first to be developed and widely used is the bleomycin model [14]. It is the best-characterized animal model due to its ability to reproduce many aspects of IPF and other fibrotic ILDs, good reproducibility and ease of induction [15]. Fibroblast-to-myofibroblast differentiation plays a key role in lung fibrosis and TGF-β remains the main hallmark implicated in myofibroblast differentiation [16]. Emerging evidence indicates that microRNAs (small, non-coding, single-stranded RNA molecules) are involved in the development of organ fibrosis and affect fibroblast-to-myofibroblast differentiation [17], including miR-101 [18], miR-9-5p [19], miR-1343 [20] and miR-27b [21], while miR-27a [22] represses myofibroblast differentiation. MicroRNAs are transcriptional regulators that could be packaged in EVs released by several cell types and participate in intercellular communication. Sato and colleagues isolated EVs of fibrocytes derived from mice models of pulmonary fibrosis involving the intratracheal administration of an adenoviral gene vector encoding active TGF-β1. They found upregulated col1a1 and miR-21-5p expression in EVs from fibrocytes and this mediated fibrogenic effects [23]. A phase-3 clinical trial testing recombinant human pentraxin 2 (an inhibitor of fibrocyte differentiation) in IPF produced the first clinical evidence that therapies targeting fibrocytes might be effective [24]. Increased expression of exosomal miR-22 in pulmonary fibrosis, modulating fibroblast-to-myofibroblast differentiation and improving lung fibrosis lesions in the bleomycin-induced mice model, were recently reported [17]. 

Human lung fibroblasts (HLF) are the most abundant cells in the lung interstitium. These mesenchymal cells produce the extracellular matrix that gives the lung its structural integrity and has a critical role in wound healing. HLFs also perform an innate immune function as sentinel cells and may play an active role in moderating the response to harmful stimuli.

Lacy and colleagues reported that activation of HLF by IL1-β inhibited TGF-β-induced myofibroblast differentiation and that EVs containing prostaglandins have antifibrotic effects on activated and naïve HLFs [25]. Guiot et al. reported that exosomal miR-142-3p is overexpressed in HLF and that alveolar epithelial cells reduce the expression of TGF-β-R1. They demonstrated that exosomes isolated from sputum macrophages have antifibrotic properties due partly to the repression of TGFβ-R1 by miR-142-3p transfer to target cells [26]. 

A functional role of bone-marrow-derived mesenchymal stem cell (BMSC)-secreted EVs has been implicated in HLF, yet their action in the treatment of IPF is not fully understood. Wan et al. investigated BMSC-derived EVs expressing miR-29b-3p in HLF in the treatment of IPF, reporting a low expression of miR-29b-3p and an overexpression of frizzled 6, α-SMA and collagen I [27]. They speculated that BMSC-derived EVs overexpressing miR-29b-3p contributed to inhibited pulmonary interstitial fibroblast proliferation, migration, invasion and differentiation. 

Another event that incites fibrosis is the increased deposition of collagen in the interstitium. This increases its tensile strength, in line with the nature of fibrotic lung disease [28]. 

Xu et al. showed elevated COL1A1 and fibronectin gene expression in experimental silicosis mice, with and without exosomes derived from human umbilical cord mesenchymal stem cells [29]. The authors reported lower collagen deposition in terms of decreased gene expression in mice treated with exosomes, suggesting that these stem cells could be part of a therapeutic strategy for silica-induced pulmonary fibrosis [29]. 

### 3.4. EVs in Bronchoalveolar Lavage from ILD Patients

Bronchoalveolar lavage is a useful biological fluid for studying inflammatory cell infiltrates and presumably also for isolating EVs to detect biomarkers of ILD [30,31,32,33,34,35]. Relatively little is yet known about EV expression and function in the local lung environment in the context of lung fibrosis and remodelling [36].

There is emerging evidence that BAL EVs play an essential role in the pathogenesis of various ILDs, including IPF [37]. Martin-Medina et al. reported increased BAL-EV function as carriers of WNT proteins and a contribution to the pathogenesis of IPF [38]. They were the first to compare EVs carrying WNT5A in BAL samples of patients with IPF, non-IPF ILD, non-ILD and healthy controls. They demonstrated that the effect of EV-associated WNT5a on fibroblast proliferation could not only be attenuated by siRNA-mediated WNT5A knockdown, but also by antibody-mediated neutralization of WNT5A on EVs [38]. 

The pathogenesis of IPF is very complex, and involves several epithelial and mesenchymal cell populations, including fibrocytes that are mesenchymal progenitor cells derived from CD14+ peripheral cells, thought to be involved in the normal wound-healing response [39]. Although the accepted hypothesis is that fibrocytes are involved in the pathogenesis of IPF, the specific mechanisms are still not clear. Moeller et al. demonstrated elevated percentages of circulating fibrocytes in IPF patients and proposed that they were predictive of early mortality in such patients [40]. The same group of researchers recently investigated miRNA-produced fibrocytes packaged into EVs. They cultured cells from human BAL samples into fibronectin-coated dishes and the fibrocytes were analysed for miRNA expression. Fibrocytes exerted pro-fibrotic effects on fibroblasts through secretion of EVs containing pro-fibrotic miRNA, and in particular they revealed higher miR-21-5p expression in fibrotic interstitial pneumonia patients than in patients with non-fibrotic disease [40]. 

In parallel to the contribution of EVs to fibrotic mechanisms, BAL EVs generated by sarcoidosis patients are reported to display pro-inflammatory effects [41,42,43,44,45]. Most studies of BAL EVs were performed in sarcoidosis patients probably due to the diagnostic purpose of bronchoscopy with BAL in the sarcoidosis diagnostic work-up.

Qazi et al. reported higher levels of exosomes in BAL from sarcoidosis patients than in BAL from healthy controls, and also higher concentrations of CD54 and MHC molecules [44]. The authors tested BAL EVs with autologous peripheral blood mononuclear cells (PBMCs) for their ability to contribute to inflammation by inducing interferon-γ (IFNγ), interleukin (IL)-13 and IL-8 [44].

In support of a direct effect on monocytes, the same group of researchers recently reported that EVs from BAL samples of sarcoidosis patients induced dose-dependent elevation of IL1-β in monocytes, confirmed also in PBMCs and enriched monocytes. Other literature reported the interesting role of the mononuclear phagocyte (MNP) system in lung disorders, including sarcoidosis, where tissue infiltration by MNPs is a hallmark [41]. 

It has been speculated that exosomes are mediators and disseminators of inflammation, opening the way for further investigations of the link between CCL2 and exosomal leukotrienes in sarcoidosis [41]. To confirm this link, Martinez-Bravo et al. analysed more than 690 proteins in exosomes from BAL samples [43]. Several of these proteins, including inflammation-associated proteins, such as leukotriene A4 hydrolase, were significantly upregulated in patients with respect to controls [43]. For the first time, they reported an abundance of vitamin D-binding protein in BAL exosomes from sarcoidosis patients, confirming their findings in plasma samples. Altered calcitriol production, parathyroid hormone (PTH) activity and sensitivity to vitamin D have been described in sarcoidosis [46]. Increased calcitriol levels promote intestinal absorption of calcium and resorption from bone. Stimulated by IFN-γ, tumour necrosis factor-α (TNF-α, IL1 and IL2), macrophages from sarcoid granulomas, can spontaneously release 1,25-dihydroxy vitamin D, further boosting calcium resorption from the gastrointestinal tract and bone, and causing hypercalcemia and hypercalciuria [47]. Since abnormal resorption is associated with bone fragility and changes in bone mineral density, calcium metabolism is an issue in sarcoidosis, especially in patients requiring prolonged steroid treatment.

MicroRNA transcriptional regulators are further markers that may have a pro-inflammatory role in pulmonary sarcoidosis. Kishore and colleagues investigated the expression of miRNA packaged in EVs during cell communication in BAL from sarcoidosis patients. Interestingly, they found an increased expression of miR-146a, miR-150 and cellular miR-21 in chest X-ray stage II of sarcoidosis and an inverse correlation with lung function parameters, and speculated on whether a typical extracellular miRNA profile might characterize distinct sarcoidosis clinical phenotypes [42].

### 3.5. EVs in Peripheral Blood from ILD Patients

A few studies analysed EVs from plasma samples, and most validated an exosome-associated biomarker in the blood of patients that may provide novel, less invasive tools for diagnosis [43]. Guiot et al. showed upregulation of miR-142-3p in the plasma of IPF patients, suggesting that this miR influences the pathogenesis of pulmonary fibrosis [26].

To our knowledge, only one paper investigates the expression of exosomal surface markers in serum from IPF patients [48]. The authors found an increase in the expression of CD19, CD8, CD69 and CD86 in such patients, suggesting and confirming the “immune profile” of IPF pathogenesis. They also reported high expression of ROR1, a key mediator of WNT5a signalling, on EVs. WNT5a signalling is a regulator of fibroblast proliferation and resistance to apoptosis, both mechanisms involved in the development and progression of lung fibrosis in IPF [48]. 

Further studies are needed to obtain data on communication signatures/exosomal profiles in serum from IPF patients. Such data could offer new insights into IPF pathogenesis and could enable the survival of IPF patients to be predicted more reliably. 

### 3.6. EVs in Sputum from ILD Patients

Induced sputum has been proposed as a useful, non-invasive method for the assessment of airway diseases, including ILDs [49,50]. Guiot and colleagues were the first to study certain markers in sputum samples from IPF patients. They found that increased expression and production of matrix metalloproteinases and interleukins may contribute to the disease [50]. They also analysed EVs from sputum and identified new diagnostic biomarkers in this non-invasive biofluid [51]. They found three miRs (miR-142-3p, miR-33a-5p and let-7d-5p) with aberrant expression in sputum-derived exosomes from IPF patients. They also observed the upregulation of miR-142-3p, directly associated with macrophage percentages in sputum of IPF patients, suggesting that sputum macrophages are a source of elevated levels of exosomal miR-142-3p in an IPF context [26]. These results were validated in airway epithelial cells and lung fibroblasts, where the overexpression of miR-142-3p, such as to reduce the expression of TGF-βR1, was found [26]. Exosomes isolated from macrophages showed antifibrotic properties due partly to the repression of TGFβ-R1 by miR-142-3p transfer to target cells.

## 4. Conclusions

Fibroblast-to-mesenchymal differentiation, the deposition of collagen and extracellular matrix and cellular senescence [52] are key mechanisms in the development and progression of IPF. EV-coupled miRNAs are important modulators of biological processes in terms of intercellular communication, as shown in pulmonary fibrosis models as well as biofluids. The emerging evidence on the role of EVs in the pathogenesis of ILD is worthy of further investigation in plasma, BAL and sputum due to the limited invasiveness of the collection of these samplings and the utility of EVs as diagnostic and theranostic markers. The emerging potential of EVs to translate effective EV-based therapies into clinical practice is of growing interest, since new therapeutic strategies for IPF patients are urgently needed.

## Figures and Tables

**Table 1:** SANRA Score for quality assessment of selected studies for the scoping review.

No.	Title, Author, Year, (Reference)	Justification of the Article’s Importance for the Readership	Statement of Concrete Aims or Formulation of Questions	Description of the Literature Search	Referencing	Scientific Reasoning	Appropriate Presentation of Data	Total Score
1	Fibrotic extracellular matrix induces release of extracellular vesicles with pro-fibrotic miRNA from fibrocytes. Sato S, Thorax, 2021 [1]	2	2	0	1	2	2	9
2	Extracellular Vesicle Surface Signatures in IPF Patients: A Multiplex Bead-Based Flow Cytometry Approach. d’Alessandro M, Cells, 2021 [2]	2	2	0	1	1	2	8
3	Exosome-Derived microRNA-22 Ameliorates Pulmonary Fibrosis by Regulating Fibroblast-to-Myofibroblast Differentiation in Vitro and in Vivo. Kuse N, J Nippon Med Sch, 2020 [3]	2	2	0	1	1	1	7
4	Exosomes derived from three-dimensional cultured human umbilical cord mesenchymal stem cells ameliorate pulmonary fibrosis in a mouse silicosis model. Xu C, Stem Cell Res Ther, 2020 [4]	1	1	0	1	2	1	6
5	Sarcoidosis exosomes stimulate monocytes to produce pro-inflammatory cytokines and CCL2. Wahlund CJE, Sci Rep, 2020 [5]	2	1	0	2	2	1	8
6	Pulmonary Extracellular Vesicles as Mediators of Local and Systemic Inflammation. Wahlund C, Front Cell Dev Biol, 2017 [6]	2	2	0	1	2	2	9
7	Macrophage-Derived Exosomes Attenuate Fibrosis in Airway Epithelial Cells through Delivery of Antifibrotic miR-142-3p. Guiot J, Thorax, 2020 [7]	2	2	0	2	2	2	10
8	Sputum Exosomes: Promising Biomarkers for Idiopathic Pulmonary Fibrosis. Njock MS, Thorax, 2019 [8]	2	1	0	2	1	2	8
9	Mesenchymal Stem Cell-Derived Extracellular Vesicles Suppress the Fibroblast Proliferation by Downregulating FZD6 Expression in Fibroblasts via Micrrna-29b-3p in Idiopathic Pulmonary Fibrosis. Wan X, J Cell Physiol, 2020 [9]	2	2	0	1	2	1	8
10	Endothelial Cell-Derived Extracellular Vesicles as Potential Biomarkers in Chronic Interstitial Lung Diseases. Neri T, Ann Clin Lab Sci, 2019 [10]	1	2	0	1	2	1	7
11	Activated Human Lung Fibroblasts Produce Extracellular Vesicles with Antifibrotic Prostaglandins. Lacy SH, Am J Respir Cell Mol Biol, 2019 [11]	2	1	0	2	2	2	9
12	Increased Extracellular Vesicles Mediate WNT5A Signaling in Idiopathic Pulmonary Fibrosis. Martin-Medina A, Am J Respir Crit Care Med, 2018 [12]	2	2	0	1	2	1	8
13	Expression Analysis of Extracellular Microrna in Bronchoalveolar Lavage Fluid from Patients with Pulmonary Sarcoidosis. Kirshore A, Respirology, 2018 [13]	2	1	0	2	1	2	8
14	Pulmonary Sarcoidosis is Associated with Exosomal Vitamin D-Binding Protein and Inflammatory Molecules. Martinez-Bravo MJ, J Allergy Clin Immunol, 2017 [14]	2	2	0	1	1	1	7
15	Procoagulant, Tissue Factor-Bearing Microparticles in Bronchoalveolar Lavage of Interstitial Lung Disease Patients: An Observational Study. Novelli F, Plos One, 2014 [15]	1	2	0	1	2	2	8
16	Proinflammatory Exosomes in Bronchoalveolar Lavage Fluid of Patients with Sarcoidosis. Qazi K, Thorax, 2010 [16]	1	1	0	17	2	2	7

## Data Availability

The data presented in this study are available on request from the corresponding author.

## Abbreviations

ILD	interstitial lung disease
IPF	idiopathic pulmonary fibrosis
EVs	extracellular vesicles
miRNA	microRNA
